# Mesoporous calcium phosphate bionanomaterials with controlled morphology by an energy‐efficient microwave method

**DOI:** 10.1002/jbm.a.35508

**Published:** 2015-06-28

**Authors:** Philip James Thomas Reardon, Jie Huang, Junwang Tang

**Affiliations:** ^1^ Department of Chemical EngineeringUCLLondonWC1E 7JEUnited Kingdom; ^2^ Department of Mechanical EngineeringUCLLondonWC1E 7JEUnited Kingdom

**Keywords:** calcium phosphate, nanobiomaterials, morphology control, mesoporous, microwave synthesis

## Abstract

Calcium phosphate nanomaterials with controllable morphology and mesostructure were synthesized via a rapid and energy efficient microwave method. An increase in aspect ratio from nanoplates to nanorods was achieved by increasing the solvent chain length, accompanied by a subsequent about 23% increase in surface area and porosity. Control of mesoporosity was also achieved by varying the synthesis time and quantity of H_2_O in the reaction solvent. Comparative studies were carried out using conventional heating (CON) and room temperature co‐precipitation (RT) methods. It was found that microwave synthesis produces nanomaterials with about 50% higher yields, 7.5/1.7 times higher surface area and 3/5 times higher pore volume than RT/CON materials respectively, as well as having a lower distribution of particle size/shape (lower standard deviation values of their dimensions). Furthermore, *in vitro* protein loading tests of microwave synthesized mesoporous calcium phosphate materials showed an enhanced loading efficiency of bovine serum albumin (3–7 times), as compared with non‐mesostructured products from room temperature precipitation, in accordance with their larger surface area and porosity. © 2015 Wiley Periodicals, Inc. J Biomed Mater Res Part A: 103A: 3781–3789, 2015.

## INTRODUCTION

Nanosized biomaterials have received a great deal of interest in recent years due to their potential to provide enhancements in crucial biomedical applications such as tissue engineering and drug delivery.^1^ Nanoparticles can be engineered to provide targeted delivery of therapeutic agents, tackling the inherent toxicity and efficacy issues associated with current systemic delivery methods.^2^ At the same time, nanomaterials have also been shown to possess superior mechanical properties and provide enhanced cell proliferation and differentiation,^3^, ^4^ meaning they have great potential as tissue engineering materials that can be combined with *in situ* drug delivery.^4^

An exciting area of biomaterial research is, therefore, optimization of particle systems for targeted and controlled delivery of drugs, and to achieve the most favorable interface with cells and tissues. Mesostructured materials have shown promise both as enhanced drug delivery agents,^5^, ^6^ and in tissue engineering.^7^ Regarding drug delivery systems, ordered mesoporous silicates have demonstrated high drug adsorption and fine control of drug release kinetics,^5^ with the matrix pore size shown to directly control the rate of release and adsorption capacity for a particular therapeutic size.^8^, ^9^ However, particle morphology and size are also critical factors which have been shown to effect drug release kinetics independent of mesopore size, for example a higher release rate was observed by Qu et al. for nanospheres compared to micron sized rods.^10^

Possessing similar composition to the mineral phase of mammalian bone, calcium phosphate (CaP) materials are of special interest as biomaterials due to their unique biocompatibility and bioactivity, which has led to their wide use in bone tissue engineering and therapeutic delivery.^11^, ^12^ Dicalcium phosphate anhydrous (DCPA), or monetite, is a bioresorbable member of this group of bioactive materials, which has been used extensively in CaP cements and recently as Ca^2+^ and PO_4_^3−^ ion releasing materials.^13^ Monetite materials have greater aqueous solubility and *in vivo* degradation rates when compared to other commonly used CaP phases,^14^ and continue to raise the interest of researchers as ideal candidates for drug delivery and bone regeneration due to their fast osteotransduction (the process whereby a substituted bone material, upon application in a defect site, is resorbed and gradually replaced by viable bone tissue) and biocompatabililty, which have recently been highlighted by a series of *in vitro*, animal and human studies.^14^, ^15^, ^16^

Given their great potential, there is currently a lot of focus on trying to control the key factors of morphology and mesoporosity of monetite and other CaP nanomaterials.^11^, ^17^ However, even though the synthesis of mesoporous silica and bioactive glass materials has been widely reported, this is not true for mesoporous calcium phosphate. Mesostructured calcium phosphate has been achieved through template‐directed growth methods, such as utilizing cationic, anionic, and catanionic surfactants.^18^, ^19^, ^20^ However, the control over particle distribution, morphology, and size of these materials was not ideal, and there remains concerns that using templating materials may result in unwanted organic residues being incorporated into the final products.^21^, ^22^, ^23^ Furthermore, these processes require lengthy reaction times of many hours, leading to difficulty in reproducibility.^22^, ^24^ Conversely, the employment of microwave dielectric heating to the solvothermal method is an emerging technology that is characterized by rapid, volumetric, selective, and differential heating, allowing for synthesis of materials with altered morphologies, increased yields, improved purity, and considerably reduced synthesis times.^25^, ^26^, ^27^ As demonstrated in our recent work, synthesis of different phases of CaP nanomaterials with plate, rod, and wire morphology is achievable via microwave synthesis.^27^, ^28^

Herein, we report a comprehensive study of structure‐controlled microwave synthesis of CaP nanomaterials by varying solvent composition and MW reaction parameters (temperature, time, and so forth). This energy efficient method was used to reproducibly manipulate the morphology and mesoporosity of CaP nanomaterials for tissue engineering/drug delivery without the use of additional templating materials, in effect producing samples that can be directly utilized *in vivo* without pretreatment. Furthermore, underlying MW‐assisted mechanisms were discussed based on diverse material characterization, and protein loading tests were undertaken to demonstrate the structure–function relationship of the materials synthesized by different methods.

## MATERIALS AND METHODS

### Materials synthesis

Material preparation consisted of a two‐step process. Initially, two 20 mL (0.17*M*) solutions of either Ca(NO_3_)_2_.4H_2_O (Sigma) or H_3_PO_4_ (Sigma) were prepared using either ethanol (EtOH), butanol (BtOH), or hexanol (HxOH), as given in Table 1. The H_3_PO_4_ solution was then added drop‐wise to the Ca(NO_3_)_2_.4H_2_O solution at room temperature and atmospheric pressure over a period of about 10 min. This relatively clear mixture (40 mL) was then transferred to a sealed 100 mL PTFE container (Easy prep, CEM) and heated by microwave irradiation, which was performed by using a predetermined temperature ramp and time program with continuous stirring (see Table 1). The microwave accelerated reaction system used (MARS, CEM) automatically adjusted the output power (up to a maximum of 400 W) in order to maintain a steady temperature and pressure profile. The ramping time was fixed at 25 min and hold times were changed between 1 and 20 min as listed in Table 1. For comparison, the same reaction mixture (using EtOH as the solvent) was also transferred to a sealed 100‐mL Teflon lined autoclave and placed in a programmable convection oven (Advantage Lab 50‐L) for 20–60 min at 200°C, as given in Table 1 (CON materials). In a separate experiment, the reaction mixture of Ca(NO_3_)_2_.4H_2_O and H_3_PO_4_ prepared using ethanol at room temperature was left under stirring in an open vessel for different time periods as given in Table 1 (RT—room temperature co‐precipitation materials with no external heating applied). The product suspension in all methods was centrifuged, whereby the solid product was separated, washed several times with DI water and EtOH, and then dried in an oven at 60°C overnight.

**Table 1:** Treatment Conditions and Properties of Various CaP Materials.

Sample Code	Solvent	Reaction Temperature (°C)	Reaction Time (min)	Specific Surface Area (m^2^ g^−1^)	Particle Size (nm)	Notes
E200‐1M	EtOH	200	1	35 ± 3.8	198 ± 52 × 68 ± 20	
E200‐5M	EtOH	200	5	60 ± 7.2	205 ± 37 × 89 ± 20	
E200	EtOH	200	20	45 ± 2.8	200 ± 57 × 107 ± 29	
B200	BtOH	200	20	51 ± 2.1	189 ± 69 × 56 ±1 8	
H200	HxOH	200	20	60 ± 5.8	135 ±54 × 26 ± 7	
E99.5W0.5	EtOH/H_2_O	200	20	32 ± 1.8	253 ± 94 × 94 ± 7	
E98W2	EtOH/H_2_O	200	20	10 ± 1.1	–	
CON‐20	EtOH	200	ca. 20	26 ± 4.1	309 ± 122 × 123 ± 49	Conventional heating
CON‐60	EtOH	200	ca. 60	29 ± 2.8	309 ± 136 × 117 ± 41	Conventional heating
RT‐60	EtOH	25	60	6 ± 1.4	146 ± 54 × 48 ±19	Not heated
RT‐180	EtOH	25	180	6 ± 0.9	157 ± 45 × 48 ± 24	Not heated

### Materials characterization

The chemical and structural composition of the samples was studied by X‐ray diffraction (XRD), attenuated total reflectance‐Fourier transform infrared spectroscopy (ATR‐FTIR), and X‐ray photoelectron spectroscopy (XPS). XRD patterns of samples were collected using a Bruker D4 Advanced powder X‐ray diffractometer as described previously,^27^, ^28^ and compared with standard spectra (JCPDS). ATR‐FTIR was performed using a Perkin–Elmer 1605 FTIR spectrometer in the frequency range 400–4000 cm^−1^. XPS measurements were performed on a Thermoscientific XPS K‐alpha surface analysis machine using an Al source. Each sample was scanned six times at different points on the surface to eliminate point error and create an average. Specific elemental peaks Ca 2p and P 2p were then identified, and analyzed further. Peak fitting and area calculations were performed on Ca 2p and P 2p high resolution scans using CasaXPS software. Peak areas were divided by their relative sensitivity factors and compared to obtain Ca/P composition data. Analysis was performed on the Thermo Advantage software. Nitrogen (N_2_) adsorption–desorption isotherms were collected in a Micromeritics TriStar gas adsorption analyzer at 77 K after degassing the samples at 140°C overnight. The surface areas of sample powders were calculated according to the Barrett–Emmett–Teller (BET) equation. The relative pressure *P*/*P*_0_ of the isotherm was studied between 0.01 and 1.0. The pore parameters were calculated from the desorption branches of the isotherm from the Barrett–Joyner–Halanda (BJH) model. The types of isotherms were evaluated according to their shape and type of hysteresis between the adsorption–desorption modes. Transmission electron microscopy (TEM) was used to analyze the particle morphology using a Jeol JEM‐1010 microscope. The mean particle dimensions were obtained by measuring 100 particles from TEM micrographs using Image J software, dimensions are quoted with standard deviation values. High resolution transmission electron microscopy (HRTEM) images were conducted on a JEOL 2010F at an accelerating voltage of 200 keV.

### Protein loading

*In vitro* protein loading experiments were conducted as previously reported.^27^ Briefly, protein loading entailed adding 0.25 g of synthesized nano calcium phosphate to a 50 mL solution (1 mg mL^−1^) of bovine serum albumin (BSA) followed by agitation at room temperature for a 24 h period. Measurements of BSA concentration were carried out using a UV–vis spectrometer (Shimadzu UV‐2550) at about 280 nm by comparison with a calibration plot (0.01–1 mg mL^−1^). The amount of loaded protein was established by measuring the difference in protein concentration in the loading medium before and after loading (depletion method). All measurements were performed in triplicate and an average value calculated.

## RESULTS AND DISCUSSION

### Influence of reaction time

**Figure 1:**
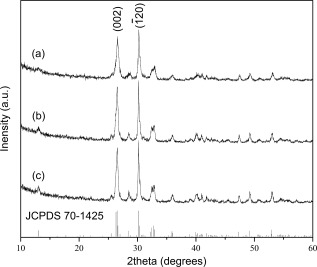
XRD patterns of materials synthesized using different periods of MW irradiation: (a) E200 (20 min), (b) E200‐5M (5 min), and (c) E200‐1M (1 min).

To investigate the influence of MW irradiation time on particle morphology and mesopore development, nanosized calcium phosphate materials were synthesized at 200°C using different reaction times with all other reaction conditions consistent (e.g., reaction temperature, ethanol as reaction solvent, and precursor stoichiometry). Figure 1 shows all peaks in the XRD patterns are consistent with crystalline monetite (JCPDS 70‐1425), with the major peaks at 2*θ* = 26.4 and 30.2, corresponding to (002) and $\left(\bar{1}20\right)$ respectively, consistent with FTIR analysis (Supporting Information Fig. S1).

**Figure 2:**
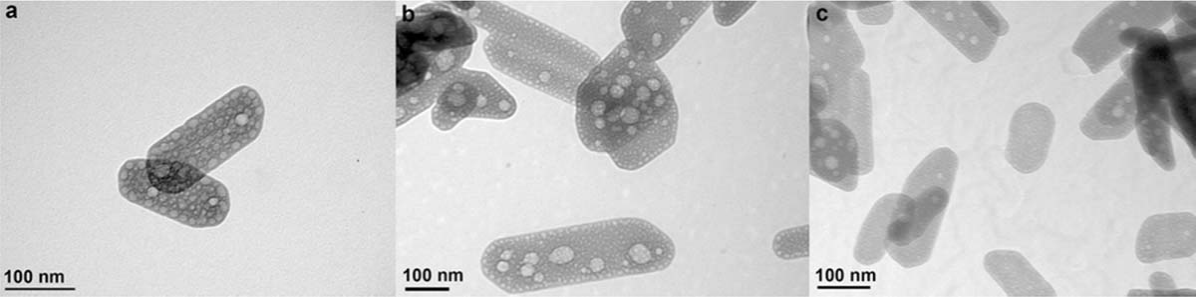
TEM micrographs showing morphology of materials synthesized using different periods of MW irradiation: (a) E200 (20 min), (b) E200‐5M (5 min), and (c) E200‐1M (1 min).

**Figure 3:**
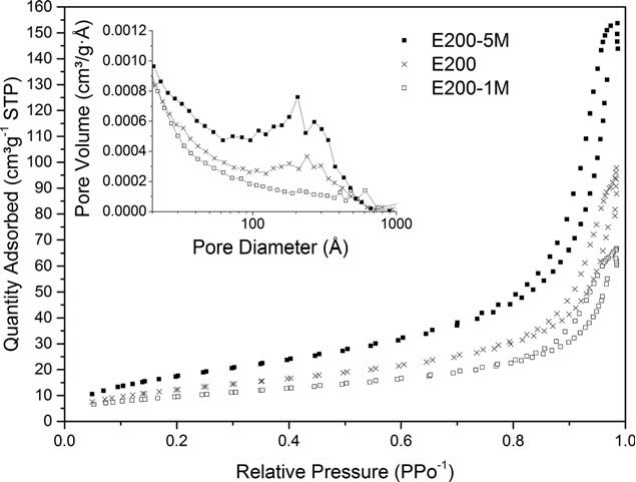
N_2_ adsorption–desorption isotherms and BJH pore size distribution (inset) for materials synthesized using different irradiation times.

TEM micrographs (Fig. 2) show that there is no significant change in the planar morphology or particle size when the synthesis/irradiation time is varied between 1 and 20 min. However, variation in mesoporosity was observed when the irradiation time was varied. Figure 3 reveals the effect of changing irradiation time on the porosity of the materials. It can be seen from the BJH pore size distribution (Fig. 3 inset) that the volume of mesopores initially increases with irradiation time, passing through a maximum at 5 min, before reducing slightly with further increase in time. Demonstrating irradiation time can be used to manipulate monetite biomaterial porosity, but not morphology.

### Influence of reaction solvent

**Figure 4:**
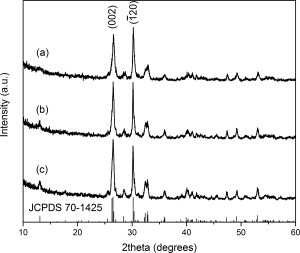
XRD patterns of materials synthesized using different alcohols as the reaction solvent: (a) E200 (EtOH), (b) B200 (BtOH), and (c) H200 (HxOH).

**Figure 5:**
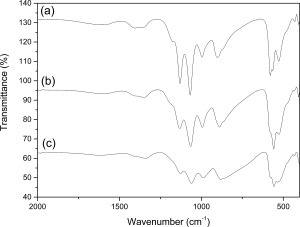
FTIR spectra of materials synthesized in different alcohols: (a) E200 (EtOH), (b) B200 (BtOH), and (c) H200 (HxOH).

The effect of solvent on the properties of monetite nanomaterials was studied using different primary alcohols as the reaction solvent. XRD, FTIR, and XPS confirmed all samples to be monetite (CaHPO_4_). Figure 4 shows that there is no change in composition when the reaction time was varied, with XRD patterns consistent with monetite (JCPDS 70‐1425). Interestingly, material H200, has a relative XRD intensity ratio between the peaks corresponding to the (002) and $\left(\bar{1}20\right)$ (selected as neutral reference) diffraction planes of about 1.13, compared to 0.87 for E200. This trend of more preferred (002) orientation suggests a preferential growth orientation with an increased expression of {00l}. FTIR bands (Fig. 5) at about 1000 to 1250 cm^−1^ are characteristic of P—O stretching vibrations. The P—O(H) stretching mode for $HPO_{4}^{2-}$ gives rise to the adsorption at 904 cm^−1^, while the P—O bending modes appear at 581 cm^−1^ and 521 cm^−1^ (*ν*′4 and *ν*′4″ respectively). The weak absorption recorded at ∼1630 cm^−1^ is due to H_2_O bending. These results are in accordance with those reported for monetite, and are consistent with no organic residues remaining in the materials.^29^

Variations in width and relative intensity of these bands (in particular peaks at ca. 520–580 and 1060–1130 cm^−1^) were observed between samples which may be due to changes in morphological structure of the monetite crystals, consistent with different growth modes observed during the TEM and XRD studies. A typical survey XPS spectrum (E200) showed peaks for each material correspond with Ca, P, O, and adventitious C (Supporting Information Fig. S2), consistent with those reported for CaP.^30^ After fitting Ca 2p and P 2p from the high resolution scans (Supporting Information Fig. S2), the areas of these peaks divided by their relative sensitivity factors results in a Ca/P ratio of 1.1, slightly above the stoichiometry of monetite bulk material (Ca/P = 1). However, experimental variation from this value is commonly reported in XPS results of many research groups.^30^ One reason suggested for experimental variations in this ratio is that dicalcium phosphate (DCP) may be affected by photodegradation, being one of the most unstable of the CaP phases, and is reported as having the greatest deviation from theoretical values.^31^ Calculated Ca/P compositions of all materials in this study are within 1 − 10% of the bulk ones, which means that the sample powders have stoichiometries similar to their bulk crystal compositions.^30^

**Figure 6:**
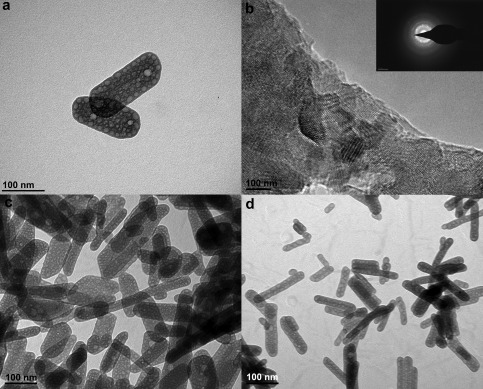
TEM micrographs of materials synthesized using different chain lengths of alcohol: (a) E200 (EtOH), (b) E200 lattice image with diffraction pattern (inset), (c) B200 (BtOH), (d) H200 (HxOH).

TEM micrographs reveal the change in material size and morphology using primary alcohols with different chain lengths (Fig. 6). The lattice structure parameters determined from high resolution TEM [Fig. 6(b)] were found to be reasonably consistent with values calculated from XRD we reported previously for microwave synthesized monetite.^28^ In comparison with the 200°C microwave synthesized nanoplate materials previously reported using EtOH as reaction solvent [Fig. 6(a)] (Table 1),^27^ the material derived from using BtOH as a reaction solvent, B200, is a mixture of nanoplates and nanorods [Fig. 6(c)] with aspect ratio about 3.4 (Table 1), while H200 synthesized using HxOH [Fig. 6(d)] comprises smaller nanorods (aspect ratio ca. 5.2) (Table 1). These observations corroborate the preferred orientation growth of monetite particles observed by XRD, showing a transition from two dimensional to one dimensional growth (along {00l}) with an increase in the solvent chain length. This demonstrates that a decrease in particle size and an increase in aspect ratio can be achieved by increasing the solvent chain length, which correlates with an increase in BET surface area from 45 ± 2.8 m^2^ g^−1^ (E200) to 60 ± 5.8 m^2^ g^−1^ (H200).

**Figure 7:**
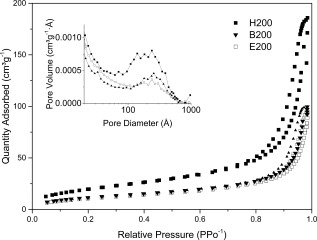
N_2_ adsorption–desorption isotherms and BJH pore size distribution (inset) for different reaction solvents.

All three materials synthesized using different chain length alcohols exhibited a type IV isotherm with H1 hysteresis loop (Fig. 7), typical of mesostructured materials with a mixture of mesopore sizes, as illustrated by the wide ranging pore size distribution (inset Fig. 7). Importantly, there is an increase in pore volume associated with longer chain length solvents (inset Fig. 7, pore volumes increases from 0.16 to 0.25 cm^3^ g^−1^), besides an increase in surface area (Table 1), indicating a facile control of the morphology, porosity, and surface area of this interesting biomaterial.

### Influence of H_2_O addition

**Figure 8:**
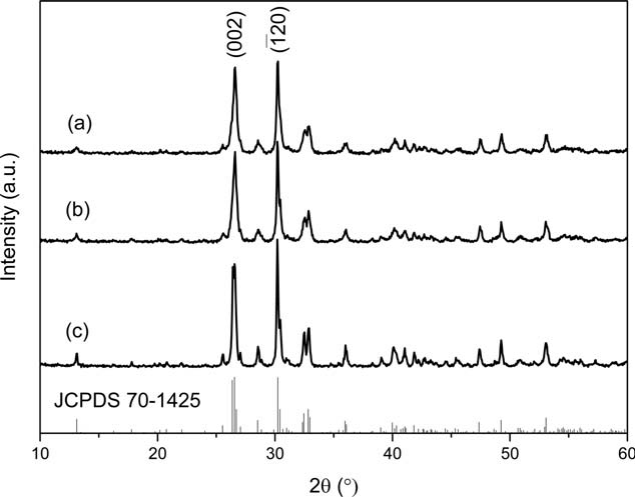
XRD patterns of materials synthesized with the addition of different volumes of H_2_O: (a) E200, (b) E99.5W0.5, and (c) E98W2.

It has been demonstrated that large amounts of water can lead to phase changes when using a dual EtOH/H_2_O solvent system in a microwave‐assisted solvothermal process.^28^ For many biomedical applications it is important to be able to produce pure phase monetite materials, so small quantities of H_2_O present in an ethanol solvothermal system (representative of typical impurity levels) was investigated and its effect on phase, porosity, and morphology was addressed. This is particularly useful for designing CaP synthesis methods involving steps under atmospheric conditions, where H_2_O vapor is present. A range of materials were synthesized using an EtOH/H_2_O solvent mixture containing 0.5–2 vol % of H_2_O. XRD patterns of all materials (Fig. 8) confirm the presence of a single monetite phase (JCPDS 70‐1425), which is consistent with FTIR spectra (Supporting Information Fig. S3), demonstrating that the expected phase can be achieved if the amount of water is <2 vol %, therefore, pure monetite can be prepared using low cost less purified alcohol solvent or with preparation steps under ambient conditions.

**Figure 9:**
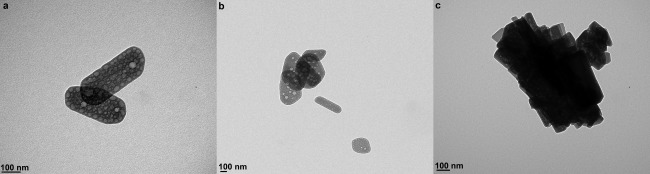
TEM micrographs of materials synthesized with the addition of different volumes of H_2_O: (a) E200, (b) E99.5W0.5, and (c) E98W2.

From the TEM micrographs (Fig. 9), it can be seen that 2 vol % of H_2_O addition (E98W2) does affect the particle arrangement, similar plate‐like particles are observed for this material but the particles appear to form micron‐sized arrangements [Fig. 9(c)]. Varying the dielectric property of the reaction medium by adding small amount of water affects not only the solubility of the dissolved solute (i.e., increasing the solubility of inorganic salt molecules), but also the colloidal interaction between solid particles (becoming weaker), leading to the increase in particle agglomeration observed when increasing the H_2_O volume in the mixed EtOH/H_2_O reaction systems.

**Figure 10:**
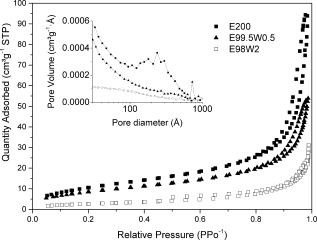
Effect of H_2_O content in the reaction solvent on the materials N_2_ adsorption–desorption isotherms and BJH pore size distributions (inset).

Importantly, N_2_ adsorption/desorption data also shows differences in the porosity of these materials. E99.5W0.5 shows a type IV (IUPAC) isotherm with a H1 hysteresis loop (Fig. 10), similar to E200 (no additional H_2_O), however there is reduced hysteresis, and an absence of mesopore peaks in the BJH pore size distribution (Fig. 10 inset). Consistent with about 50% reduction in BJH total pore volume from 0.16 to 0.085 cm^3^ g^−1^, E98W2 exhibits a type II isotherm with a type H3 hysteresis loop (Fig. 10), characteristic of aggregated plate‐like particles with nonporous or macroporous adsorbents (E99W1 is similar to E98W2, not shown here), consistent with the absence of peaks in the pore size distribution (Fig. 10 inset).^32^ Therefore, an increase in H_2_O vol % in the reaction solvent correlates with a reduction in porosity of these materials (Fig. 10) and their surface area (Table 1), demonstrating further that solvent composition change can be used to control the mesoporosity of the resultant nanomaterials.

Generally, the porosity of inorganic nanoparticles is controlled by the presence of templating agents such as polymers and surfactants. In the present system, rapid energy input and high‐pressure conditions fostered through microwave heating, coupled with the use of organic solvents, produced mesoporous CaP materials. Alcohols such as ethanol are known to interact strongly via hydrogen bonding with phosphate groups, and have an amphiphilic nature where strong and specific interactions can exist between hydrophobic parts of the solute molecules.^33^ We propose that the organic solvent molecules may cause pores or defects as material forms around them, which then agglomerate forming mesopores of varying size. Therefore, the interaction between both solvent molecules and solvent‐CaP surfaces is critical for pore formation, both of which are dependent on the composition of the solvent, which is consistent with changes in mesopore volume observed with both water addition and changes in alcohol chain length (Figs. 7 and 10).

### Effect of MW irradiation

**Figure 11:**
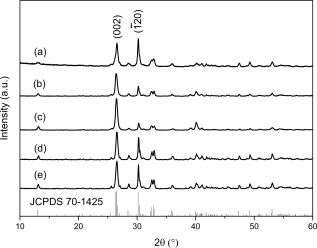
XRD patterns comparing materials synthesized without irradiation, or using conventional heating methodology to that using MW‐assisted synthesis: (a) E200, (b) RT‐60, (c) RT‐180, (d) CON‐20, (e) CON‐60.

**Figure 12:**
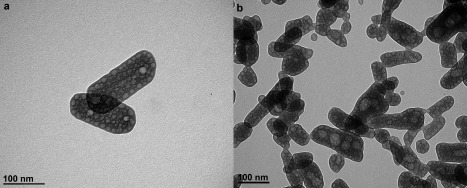
TEM micrographs materials synthesized in the presence and absence of microwave irradiation: (a) E200 and (b) RT‐60.

Material synthesis was also carried out in the absence of elevated temperature or pressure conditions, and using a conventional heating source. This approach was used to investigate the influence of both microwave irradiation and solvothermal conditions on material characteristics. The materials RT‐60 and RT‐180 formed after 1 and 3 h stirring at room temperature and atmospheric pressure (using precursor concentrations and solvent consistent with E200 preparation) have XRD patterns (Fig. 11) consistent with that of crystalline monetite (JCPDS‐70‐1425). This correlates with the study by Tas, who reported monetite synthesis at room temperature in EtOH solutions.^34^ Similarly, the materials produced by changing the heating source to a conventional one (CON‐20 and CON‐60) whilst keeping all other parameters consistent with E200 preparation (e.g., ethanol solvent, 200°C) are crystalline monetite (JCPDS‐70‐1425) (Fig. 11). Therefore, implying the material phase is controlled by the precursor compounds rather than the preparation method. However, ratios between the intensities of the peaks corresponding to the (002) and $\left(\bar{1}20\right)$ diffraction planes are higher for all the materials yielded under room temperature and atmospheric pressure (ratios of ca. 2.3 and 4.6 for RT‐60 and RT‐180 respectively), compared to E200 (an equivalent peak ratio of 1.04) (Fig. 11) produced using similar a solvent and precursors but via microwave synthesis (E200). Similarly, XRD patterns of all materials synthesized for different time periods using a conventional heating source (CON‐20, and CON‐60) have peak intensity ratios corresponding to the (002) and $\left(\bar{1}20\right)$ diffraction planes that are higher (1.12, and 1.16 for CON‐20 and CON‐60 respectively) than the equivalent microwave synthesized materials. Implying that the materials synthesized both at room temperature and using conventionally heated synthesis are of structure with higher {00l} preference in comparison to microwave synthesized materials, which correlates with the increase in one dimensional growth observed for these materials in TEM analysis (Fig. 12 and Supporting Information Figs. S4, S5 and Table 1).

TEM micrographs (Fig. 12 and Supporting Information Fig. S4) reveal that all materials synthesised for different times under atmospherics conditions and at room temperature (RT‐60 and RT‐180) have smaller and very inconsistent dimensions compared to the microwave synthesized materials, for example the standard deviation values of the dimensions of RT‐60 are about 50% higher than the equivalent microwave synthesised material‐E200 (Table 1). On the other hand, material synthesized using conventional heating (CON‐20) has a similar nanoplate morphology to the equivalent microwave synthesized material (E200), but are about 1.5 times larger than E200 (Supporting Information Fig. S5) and have a larger aspect ratio (Table 1). Importantly, the conventionally heated materials are less consistent in size and morphology, with higher standard deviation values of their dimensions (larger distribution of shape/size) (Table 1). Microwave irradiation is characterized by rapid and controlled volumetric heating. Therefore, the narrower distribution of size and morphology can be attributed to the special MW heating interaction. Furthermore, there was also about 50% reduction in product yield by the room temperature co‐precipitation synthesis, even when synthesis time was extended up to 5 h, demonstrating the high yielding capacity of using microwave synthesis.

**Figure 13:**
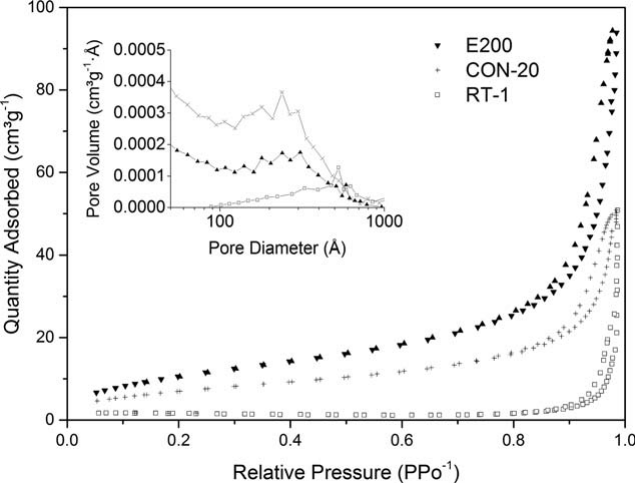
Adsorption–desorption isotherms and BJH pore size distribution (inset) for materials without irradiation, or using conventional heating methodology to replace MW‐assisted synthesis.

The materials synthesised using different synthesis methodologies were further characterized for their mesoporous structure. Figure 13 plots N_2_ adsorption/desorption isotherms of selected materials produced using the similar solvent, precursors, and total reaction time but different synthesis methods: MW‐assisted solvothermal (E200), conventional solvothermal (CON‐20), and room temperature co‐precipitation (RT‐60) synthesis. CON‐20 exhibited an IUPAC type IV isotherm with a H1 hysteresis loop type, like E200, characteristic of a mesostructured material. On the other hand, RT‐60 displayed a type II isotherm, representative of a non‐porous adsorbent.^35^ Importantly, the mesopore volume is higher for E200 (0.16 cm^3^ g^−1^) than CON‐20 (0.08 cm^3^ g^−1^) as demonstrated by the BJH pore size distribution (inset Fig. 13), consistent with a reduction in surface area from 45 to 26 m^2^ g^−1^. This demonstrates that microwave synthesis is beneficial for mesoporous CaP nanomaterials.

Yuan et al. described the development of vesicular mesopores due to electron irradiation, describing a restructuring of the CaP mesophase due to the rapid temperature increase of electron‐irradiation.^36^ A similar development and coalescence of pore‐structures into large mesopores was also observed from TEM analysis in this study. A mechanism of pore development under electron irradiation can explain why mesopores were observed in the TEM micrographs of the RT materials (Fig. 12 and Supporting Information Fig. S4), but were not recorded during N_2_ adsorption/desorption measurements (Fig. 13), and reveals that heating conditions can influence the formation of mesopores. Microwave irradiation is characterized by the direct coupling of irradiation with solvent molecules, producing rapid and controlled volumetric heating with high local temperatures. Therefore this demonstrates that formation of mesoporous CaP is controlled by both the solvent and heating methodology.

### Protein loading

**Figure 14:**
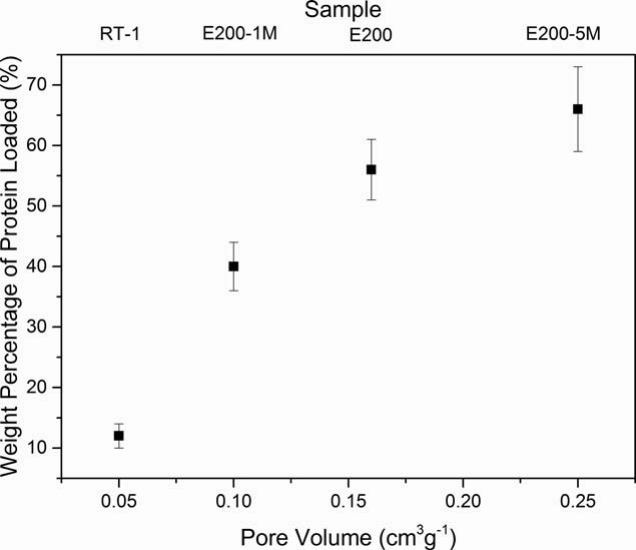
Influence of monetite pore volume on the BSA loading efficiency.

The loading efficiency of porous nanoplate materials with therapeutic drugs and proteins was studied by loading selected materials E200‐1M, E200‐5M, and E200 with BSA. BSA was loaded into materials with controlled mesoporosity (E200‐1M, E200‐5M, and E200 with pore volumes of 0.1, 0.16, and 0.25 cm^3^ g^−1^ respectively) and a non‐porous monetite material RT‐60 (pore volume—0.25 cm^3^ g^−1^) to ascertain differences in their protein loading efficiencies. The results (Fig. 14) show that mesoporous nanoplate materials E200, E200‐1M, and E200‐5M, due to their higher porosity/pore volume, have superior protein loading properties compared with the non‐porous RT material, with a 5.5 times increase in protein loading between the non‐porous RT‐60 material and the most porous monetite material (E200‐5M). Furthermore, a trend of increased BSA loading with increased porosity was observed for the mesoporous nanoplate materials (Fig. 14), equivalent to about 40% increase in loading wt % from E200‐1M (pore volume—0.1 cm^3^ g^−1^) to E200‐5M (pore volume—0.25 cm^3^ g^−1^), suggesting a direct correlation between mesoporosity and protein loading. Based on these findings, an extended study on loading different proteins is underway to further explore this trend.

## CONCLUSION

A systematic study investigating the morphologies, porosity, crystallinity, and surface area of biocompatible monetite materials has been carried out by rapid microwave solvothermal synthesis in the absence of organic templates. We observed that the most important characteristic affecting their growth in such a system was the choice of solvent. Due to the differential nature of MW heating, about 40% reduction in particle size and a change from nanoplate to nanorod morphology was observed with an increase in solvent chain length from C_2_–C_6_. Increasing mesoporosity was also observed with increasing solvent chain length. Furthermore, choice of solvent combined with high temperature and pressure conditions fostered through MW‐assisted solvothermal synthesis was found to be crucial for controlling mesopore formation. MW irradiation time was found to control the porosity of CaP materials but not morphology. Also, the presence of small H_2_O volumes in the reaction solvent drastically reduced the BJH total pore volume by about 50% and caused greater agglomeration but maintained a pure monetite phase. Compared with materials synthesized by room temperature co‐precipitation, MW synthesis gave 50% higher yields and much higher surface areas and porosity. Importantly, when compared to products by a conventionally heated system, MW synthesized materials had higher surface areas, smaller particle sizes, and a narrower size/shape distribution. MW synthesized mesoporous monetite demonstrated superior loading of BSA protein, leading to a 5.5‐fold increase compared with non‐porous monetite prepared under room temperature and atmospheric pressure conditions.

To summarize, we present a scalable, robust route to synthesise high purity, porous monetite biomaterials via rapid microwave synthesis. This represents a viable and reproducible alternative to conventional heating methods for the synthesis of well‐defined porous CaP structures without the need for toxic organic templates.

## Supporting information

Supporting Information
