# Supplementary material for: Mesoporous calcium phosphate bionanomaterials with controlled morphology by an energy‐efficient microwave method
Source: J Biomed Mater Res A. 2015 Jun 28;103(12):3781–9. doi: 10.1002/jbm.a.35508 (PMC5102653; doi:10.1002/jbm.a.35508)
Supplement: Supplementary file 1 — Supporting Information [file JBM-103-3781-s001.doc]

Supporting Information

# Mesoporous Calcium Phosphate Bionanomaterials with Controlled Morphology by an Energy-Efficient Microwave Method

Philip James Thomas Reardon, Jie Huang and Junwang Tang*

(a)

(b)

(c)

**Figure S1.** FTIR spectra of materials synthesised using different periods of MW irradiation; (a) E200, (b) E200-5M and (c) E200-1M.

**Figure S2.** Top: XPS survey spectrum, Bottom: Ca 2p and P 2p XPS high resolution scan for E200.

(a)

(b)

(c)

**Figure S3**. FTIR spectra of materials synthesised with the addition of different volumes of H_2_O; (a) E200 (b) E99.5W0.5 and (c) E98W2.


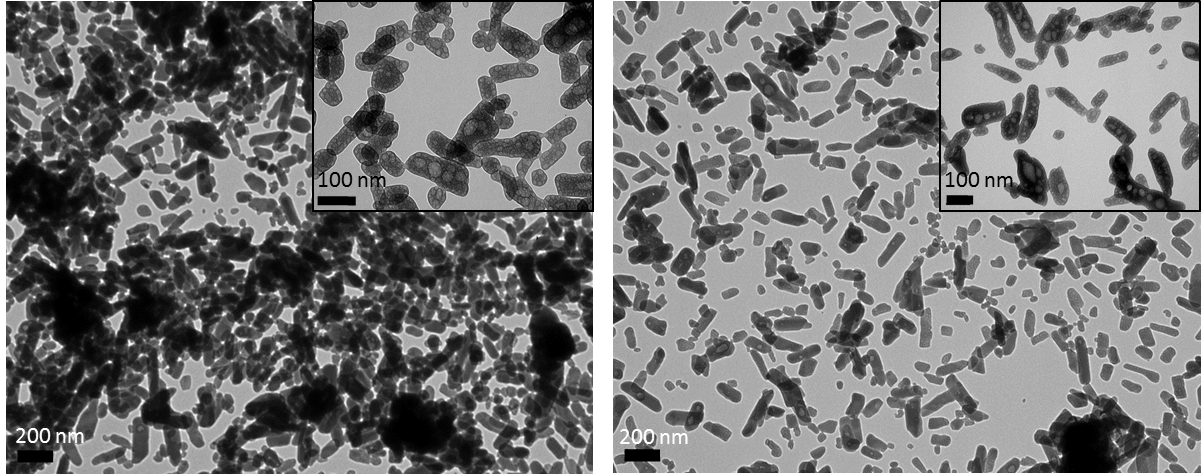


a

b

**Figure S4**. TEM micrographs of monetite materials synthesised for: (a) one hour (RT-60), and (b) 3 hours (RT-180) at room temperature and atmospheric pressure.


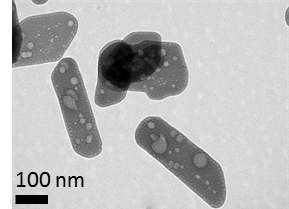


**Figure S5**. TEM micrographs of monetite materials synthesised at 200 °C in the absence of microwave irradiation (CON-20)
